# Novel non-antibiotic triple therapy for *Helicobacter pylori*-positive functional dyspepsia patients resistant to conventional antibiotic treatments: an exploratory pilot study

**DOI:** 10.3389/fmed.2026.1759043

**Published:** 2026-02-11

**Authors:** Canyu Zhan, Yurong Huang, Zhengyi Yang, Hua Wu, Juan Zheng, Wangliu Yang, Junjie Rao, Gengqing Song, Jie Yang

**Affiliations:** 1 Department of Gastroenterology, The Affiliated Hospital of Guizhou Medical University, Guiyang, China; 2 Department of Gastroenterology, Xingyi People's Hospital, Xingyi, China; 3 Department of Gastroenterology, Liupanshui Municipal People's Hospital, Liupanshui, China; 4 Department of Gastroenterology and Hepatology, Metrohealth Medical Center, Case Western Reserve University, Cleveland, OH, United States

**Keywords:** berberine hydrochloride, *Clostridium butyricum*, functional dyspepsia, *Helicobacter pylori*, non-antibiotic therapy, Weisu granules

## Abstract

**Purpose:**

The rising antibiotic resistance has significantly reduced the efficacy of standard bismuth-based quadruple therapy for *Helicobacter pylori* (*H. pylori*) infections, particularly in patients with multiple eradication failures. This study evaluates a novel non-antibiotic triple therapy comprising Weisu granules, berberine hydrochloride, and Bio-Three—a probiotic formulation containing *Clostridium butyricum* TO-A, *Enterococcus faecalis* T-110, and *Bacillus mesentericus* TO-A—in treating *H. pylori*-positive functional dyspepsia (FD) patients resistant to conventional antibiotic treatments.

**Patients and methods:**

A two-center retrospective analysis involved 48 FD patients who had previously failed at least two *H. pylori* eradication therapies. Participants underwent a 14-day course of non-antibiotic therapy, with primary endpoints being *H. pylori* eradication rate, assessed via 14C urea breath test, and secondary endpoints including symptom relief and adverse reactions.

**Results:**

Successful *H. pylori* eradication was achieved in 72.9% (35/48) of patients. Symptom relief was observed in 77.08% of cases, with 43.75% achieving effective improvement and 33.33% marked improvement. Adverse reactions were mild, occurring in 8.3% (4/48) of patients, including abdominal distension, dry mouth, and nausea, all resolving spontaneously. The compliance rate was high at 91.67%.

**Conclusion:**

This study provides preliminary evidence supporting the use of non-antibiotic triple therapy as an alternative for managing *H. pylori* infections in FD patients resistant to conventional antibiotic treatments, demonstrating notable eradication rates and symptom relief with minimal adverse effects. Further research is warranted to explore its mechanisms, long-term efficacy, and potential integration into existing treatment paradigms.

## Introduction

*Helicobacter pylori* (*H. pylori*) is a widespread pathogen that infects approximately half of the global population (1). Although improved sanitary conditions have reduced the incidence of *H. pylori* infection (2), it remains a significant cause of various gastrointestinal and extraintestinal disorders, particularly as a major risk factor for gastric cancer. Thus, successful eradication of *H. pylori* is crucial for reducing gastric cancer risk (2, 3).

Functional Dyspepsia (FD) is classified as a brain-gut interaction disorder (DGBI), characterized by upper abdominal pain or burning sensation, early satiety, and postprandial fullness. Its etiology is multifactorial, involving *H. pylori* infection, dietary habits, gastric physiological abnormalities, psychological factors, and duodenal inflammation (4). Animal models have shown that *H. pylori* infection induces gastric sensorimotor alterations similar to those observed in FD patients, which can normalize after *H. pylori* eradication (5). Meta-analyses have demonstrated the superior efficacy of *H. pylori* eradication over other therapeutic modalities in ameliorating FD symptoms (6, 7).

Currently, bismuth quadruple therapy remains the primary first-line regimen for *H. pylori* eradication (1). However, escalating resistance rates and non-standardized treatment approaches have reduced the success rates of initial eradication, leading to a higher prevalence of treatment failures. The definition of refractory *H. pylori* infection varies: the sixth edition of the Chinese Consensus defines it as persistence after two or more standard eradication treatments (8), while the American College of Gastroenterology (ACG) guidelines consider failure of the first-line treatment alone as refractory (9). Although guidelines recommend bismuth quadruple therapy as an empirical salvage regimen and advocate for antibiotic sensitivity testing in salvage therapy (8, 10, 11), repeated treatment failures raise concerns about multidrug resistance, secondary antibiotic resistance, and increased adverse drug reactions. Despite the ideal utility of antibiotic sensitivity testing, its widespread clinical application is hindered by prolonged turnaround times and high costs. Consequently, some patients continue to experience treatment failures despite the use of sensitive antibiotics. Therefore, enhancing the efficacy of salvage treatment represents a critical clinical challenge in the management of *H. pylori* infections.

China’s latest guidelines recommend combining bismuth quadruple therapy with traditional Chinese medicine or substituting traditional Chinese medicine for bismuth to enhance the eradication rate of *H. pylori*, alleviate patient symptoms, and reduce adverse reactions (8). Weisu Granules is a standardized herbal formulation primarily utilized for the treatment of functional dyspepsia and chronic gastritis (12). The primary ingredients of Weisu granules include *Perilla frutescens* (Zi Su Geng), *Cyperus rotundus* (Xiang Fu), *Citrus reticulata* (Chen Pi), *Citri Sarcodactylis Fructus* (Foshou), *Aurantii Fructus* (Zhi Ke), *Areca catechu* (Bing Lang), and *Gallus gallus domesticus* (Ji Nei Jin). The active constituents of Weisu Granules, particularly flavonoids like hesperidin and volatile oils, have been shown to modulate the brain-gut axis and improve gastric compliance (12). In the context of *H. pylori* infection, these compounds may help restore the gastric mucosal barrier and alleviate the low-grade inflammation that persists even after the pathogen is suppressed. Recent research indicates that combining Weisu granules with triple therapy effectively increases *H. pylori* eradication rates (93.88% vs. 73.47%) and significantly reduces gastrointestinal symptom scores and the incidence of adverse reactions (13).

Berberine, another Chinese herbal medicine, is an isoquinoline alkaloid derived from Coptis chinensis and other Berberis plants (14). Studies have shown that berberine derivatives can penetrate the mucus layer and effectively eradicate *H. pylori* biofilms (15). Clinical trials have indicated that triple therapy containing berberine, amoxicillin, and rabeprazole is non-inferior to bismuth quadruple therapy (eradication rate by PP analysis: 83.6% vs. 85.1%) (16).

Probiotics can alleviate clinical symptoms of *H. pylori* infection, improve eradication efficacy, and reduce adverse reactions (17). Bio-Three is a standardized triple-strain probiotic preparation consisting of *Enterococcus faecalis* T-110 (lactic acid bacteria), *Clostridium butyricum* TO-A (butyric acid-producing bacteria), and *Bacillus mesentericus* TO-A (saccharifying bacteria). Evidence suggests that *Bacillus* probiotics, including *B. mesentericus* (a key constituent of Bio-Three), exert their therapeutic effects by competitively inhibiting pathogens, reinforcing the mucosal barrier, and modulating the host’s immune response (18). These probiotics produce essential metabolites, such as short-chain fatty acids and antimicrobial peptides, which create an unfavorable gastric environment for *H. pylori* survival and facilitate its clearance (18). *Clostridium butyricum* also exhibits broad efficacy against *Clostridioides difficile*, *H. pylori*, and antibiotic-resistant *Escherichia coli*, serving as a potential antimicrobial-resistance (AMR) countermeasure (19). Treatment with *Clostridium butyricum*, *Bacillus coagulans*, or a combination of both effectively inhibits *H. pylori* and minimizes adverse reactions, reducing antibiotic resistance burdens (20).

For patients with treatment-resistant FD who have undergone multiple antibiotic regimens, the efficacy of non-antibiotic therapies remains unclear. Limited research exists on non-antibiotic approaches for *H. pylori* eradication failure, particularly complete non-antibiotic combinations. Therefore, a retrospective analysis was conducted on FD patients with prior *H. pylori* eradication failures who opted for a 14-day non-antibiotic therapy. The study aimed to assess the efficacy and safety of non-antibiotic triple therapy comprising Weisu granules, berberine hydrochloride, and Bio-Three (containing *Clostridium butyricum* TO-A*, Enterococcus faecalis* T-110*, and Bacillus mesentericus* TO-A) as a rescue treatment for *H. pylori* infection in FD patients.

## Methods

### Research design and ethics

This real-world, two-center, retrospective, single-arm study was conducted from March 2022 to February 2023 at the outpatient departments of The First Affiliated Hospital of Guizhou Medical University and Liupanshui Municipal People’s Hospital, Guizhou Province, China. This study utilized a retrospective design to evaluate patients diagnosed with FD and multiple (two or more) *H. pylori* eradication failures. These patients had previously provided informed consent for clinical treatment to receive a non-antibiotic salvage regimen consisting of Weisu granules, berberine hydrochloride, and Bio-Three. Patient data, including past medical histories, esophagogastroduodenoscopy (EGD) results, general information, personal histories, and follow-up treatment data, were recorded. Ethical approval for this study was obtained from the participating hospitals (Approval No: LPSSYY-2023-103). Due to the retrospective nature of this study, the requirement of study-specific informed consent for data analysis was waived by the Institutional Review Board, provided that all patient identifiers were strictly anonymized.

### Inclusion and exclusion criteria

Inclusion criteria: (1) Age ≥ 18 years, irrespective of gender; (2) Patients with FD, diagnosed according to the Rome IV criteria; (3) Positive for *H. pylori* infection (such as via ^14^C/^13^C urea breath test); (4) Experienced two or more failed attempts at *H. pylori* eradication; (5) Undergoing non-antibiotic triple therapy for *H. pylori* in this study.

Exclusion criteria: (1) Use of proton pump inhibitors (PPI), potassium-competitive acid blockers (P-CAB), bismuth, antibiotics, other probiotics, or traditional Chinese herbal medicine with anti-*H. pylori* properties within 1 month after *H. pylori* eradication therapy; (2) Known drug allergies; (3) EGD findings obtained during baseline screening (within 6 months before enrollment) indicating acute peptic ulcer, severe esophagitis (grade C or D), or upper gastrointestinal tumor; (4) Loss to follow-up; (5) History of subtotal or total gastrectomy.

### Diagnosis and treatment of *Helicobacter pylori* infection

The ^14^C/^13^C urea breath test (^14^C/^13^C-UBT) was conducted using specific analyzers: the ^14^C-UBT analyzer (HUBT-20P, Shenzhen Zhonghe Haidewei Biological Technology Co., Ltd., China) and the ^13^C-UBT analyzer (RICHEN HEALTH SCIENCE Shanghai Licheng Nutrition Technology Co., Ltd., China), to confirm *H. pylori* infection.

The intervention consisted of a non-antibiotic triple therapy administered for 14 days. The regimen included Weisu granules (Yangzijiang Pharmaceutical Group Jiangsu Pharmaceutical Co., Ltd., Batch No.: Z10950007, China), berberine hydrochloride (Beijing Great Wall Pharmaceutical Co., Ltd., Batch No.: H11020277, China), and Bio-Three (Huizhou Jiuhui Pharmaceutical Co., Ltd., Batch No.: SJ20160008, China). Detailed dosing specifications and total quantities administered per patient are summarized in Supplementary Table S1. Specifically, Weisu Granules 5 g (1 bag) were administered three times daily (t.i.d.), totaling 42 bags per treatment course. Berberine Hydrochloride 0.3 g (three 0.1 g tablets) was administered t.i.d., totaling 126 tablets per course. Bio-Three 400 mg (two 200 mg tablets) was administered t.i.d., totaling 84 tablets per course. All medications were administered orally. To ensure high treatment adherence, the total 14-day supply was dispensed to each participant at the baseline visit, and patients were instructed to return any unused medication or empty packaging at the follow-up appointment.

### Observational indicators of study design

The primary endpoint was the eradication rate of *H. pylori* in non-antibiotic triple therapy. This was assessed at least 1 month post-treatment using the ^14^C-UBT or ^13^C-UBT. Negative test results indicated successful eradication, while positive results indicated treatment failure.

Secondary endpoints included symptom relief rates, adverse events, and treatment compliance. Symptom severity was evaluated before and 1 month post-treatment using the Functional Dyspepsia Rating Scale (FDDQL), which assesses symptoms such as epigastric pain, postprandial discomfort, and decreased appetite. Symptoms were scored from 0 (no symptoms) to 4 (severe symptoms, significantly impacting daily life). Therapeutic responses were categorized as: <50% improvement (ineffective), 50–79% improvement (effective), and ≥80% improvement (markedly effective).

The improvement rate (IR) of clinical symptoms was calculated using the formula: IR (%) = [(total score of clinical symptoms before treatment-total score of clinical symptoms after treatment) / total score of clinical symptoms before treatment] × 100%. Markedly effective and effective responses were combined to form the total clinical effectiveness.

Compliance was determined by comparing the actual medication intake with the prescribed dosage. Good compliance was defined as ≥80% of the prescribed medication intake, and poor compliance as <80%.

### Statistical analysis

Statistical analysis was conducted using SPSS 26.0. The normality of measurement data was assessed using the Kolmogorov–Smirnov test. Measurement data following a normal distribution were presented as mean ± standard deviation (x ± s). Chi-square test, continuous correction chi-square test, or Fisher’s exact probability method were employed for comparison of count data. When the measurement data from two groups were normally distributed and had equal variances, as confirmed by the K-S test, the independent-samples *t*-test was applied. A significance level of *p* < 0.05 denoted a statistically significant difference.

## Results

### Patient demographics and clinical characteristics

A total of 56 patients were assessed for eligibility. During baseline screening, 5 patients were excluded because of EGD findings of acute peptic ulcers, which precluded a diagnosis of FD per Rome IV criteria. The remaining 51 patients were enrolled and initiated the non-antibiotic treatment protocol. During the 14-day study period, 3 patients were lost to follow-up due to an inability to establish telephone contact. A final cohort of 48 patients (19 males, 29 females; mean age 48.57 ± 11.14 years) completed the protocol and were included in the efficacy analysis.

All patients had experienced treatment failure at least twice, with 70.83% (34/48) failing twice and 29.17% (14/48) failing three times. Previous treatments included bismuth quadruple therapy, combining PPIs with amoxicillin and clarithromycin, or PPIs with amoxicillin and furazolidone for double failures. Fourteen patients who failed eradication after three attempts had previously received a 14-day salvage bismuth quadruple therapy consisting of esomeprazole (40 mg b.i.d.), bismuth potassium citrate (0.1 g t.i.d.), minocycline (0.1 g b.i.d.), and cefuroxime (0.5 g b.i.d.). All medications were administered 30 min before meals (PPI and Bismuth) or 30 min after meals (Antibiotics).

Table 1 presents the demographic and clinical characteristics of the patients. Among them, 3 patients had a family history of gastric cancer, 7 patients had a history of hypertension, and 1 patient had a history of hypertension complicated by cerebrovascular accident. The detailed demographic and clinical characteristics are summarized in Table 1.

**Table 1:** Baseline characteristics of included patients.

Characteristics	Total (*n* = 48)	Successful eradication (*n* = 35)	Eradication failure (*n* = 13)	*t*/*X*^2^	*P*-value
Age (mean ± SD)	48.57 ± 11.14	48.29 ± 11.68	49.38 ± 9.36	−0.305	0.762
Gender					
Female	29 (60.4%)	22 (62.9)	10 (76.9)	0.844	0.358
Male	19 (39.6%)	13 (37.1)	3 (23.1)		
BMI (kg/m^2^)	22.09 ± 2.82	22.02 ± 2.93	22.27 ± 2.59	−0.267	0.790
Ethnic					
Han	46 (95.8)	34 (97.1)	12 (92.3)	2.813	0.473
Chuanqing	1 (2.1)	0 (0.0)	1 (7.7)		
Yi	1 (2.1)	1 (2.9)	0 (0.0)		
Smoking					
No	34 (70.8)	25 (71.4)	9 (69.2)	0.008	0.927
Yes	14 (29.2)	10 (28.6)	4 (30.8)		
Alcohol use					
No	32 (66.7)	22 (62.9)	10 (76.9)	0.844	0.358
Yes	16 (33.3)	13 (37.1)	3 (23.1)		
Past medication history					
Hypertension	7 (14.6)	5 (14.3)	2 (15.4)	0.009	0.924
Hypertension and cerebrovascular	1 (2.1)	1 (2.9)	0 (0.0)	0.379	0.538
Family history of gastric cancer	3 (6.3)	3 (8.6)	0 (0.0)	1.189	0.276
Previous treatment failures					
2 times	34 (70.83%)	26 (74.3)	8 (61.5)	0.746	0.388
3 times	14 (29.17%)	9 (25.7)	5 (38.5)		
Education level					
Middle school and below	20 (41.7)	14 (40.0)	6 (46.2)	0.614	0.736
High school	19 (39.6)	15 (42.9)	4 (30.8)		
Bachelor’s degree and above	9 (18.7)	6 (17.1)	3 (23.1)		

Data are presented as *n* (%) or mean ± SD. BMI, body mass index; SD, standard deviation.

### *Helicobacter pylori* eradication rate and symptom relief

As shown in Table 2, among the 48 patients who completed the study, 35 achieved successful *H. pylori* eradication, while 13 did not, resulting in an overall eradication rate of 72.9%. Specifically, among the 34 patients with 2 prior treatment failures, 26 achieved successful eradication, yielding a 76.5% eradication rate. Among the 14 patients with 3 prior eradication failures, 9 achieved successful eradication, yielding a 64.3% success rate (Table 2). Analysis of demographic and clinical data revealed no significant risk factors associated with eradication failure, including gender, smoking or drinking habits, body mass index (BMI), number of previous treatments, and family history of gastric cancer (Table 1).

**Table 2:** Eradication rate and symptom relief rate.

	Total (*n* = 48)	Failed 2 times (*n* = 34)	Failed 3 times (*n* = 14)	*t*/*X*^2^	*P*-value
Eradication rate	72.9% (35/48)	76.5% (26/34)	64.3% (9/14)	0.256	0.613
Symptom score before treatment	2.75 ± 1.02	2.76 ± 1.13	2.71 ± 0.73	0.154	0.878
Symptoms score after treatment	1.06 ± 0.95	1.09 ± 1.03	1 ± 0.78	0.288	0.774
Degree of symptom relief					
Markedly effective	16 (33.33)	12 (35.29)	4 (28.57)	0.414	0.813
Effective	21 (43.75)	15 (44.12)	6 (42.86)		
Ineffective	11 (22.92)	7 (20.59)	4 (28.57)		
Symptom remission rate	77.08%	79.41%	71.43%	1.718	0.19

Data are presented as *n* (%) or mean ± SD.

The combined symptom relief rate (effective + markedly effective) reached 77.08%. Specifically, 43.75% (21/48) of patients experienced effective symptom improvement (50 to 79%), while 33.33% (16/48) achieved marked effectiveness (over 80%). Conversely, 22.92% (11/48) of the patients showed ineffectiveness (less than 50% improvement).

### Correlation analysis between symptom improvement and *Helicobacter pylori* eradication

**Figure 1:**
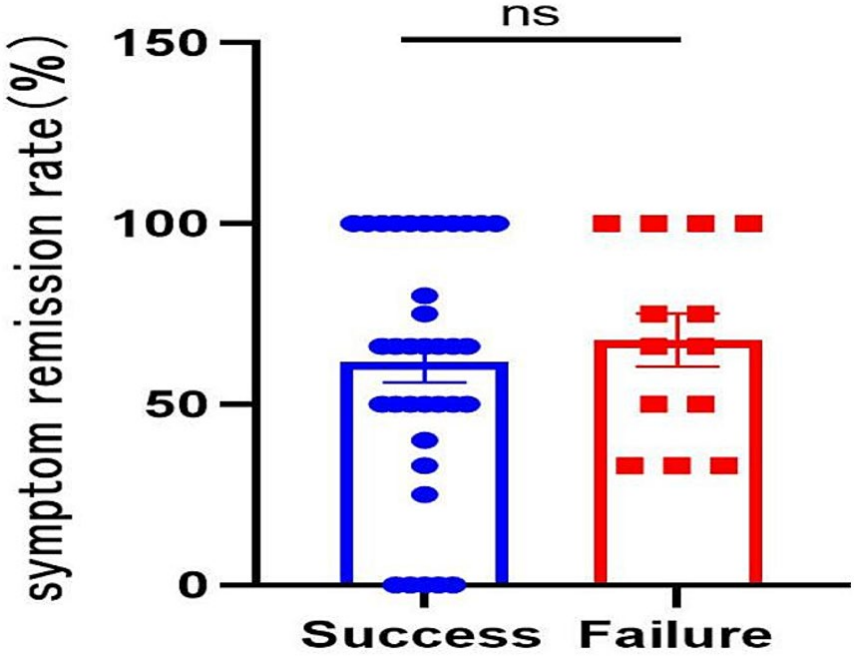
Correlation between *Helicobacter pylori* eradication and symptom improvement.

As illustrated in Table 3 and Figure 1, clinical symptom improvement was observed across the entire study population. However, a comparative analysis revealed no significant correlation between the successful *H. pylori* eradication and the magnitude of symptom relief. Specifically, patients who failed to achieve eradication experienced symptomatic improvements comparable to those who were successfully eradicated. This suggests that the clinical benefits of the non-antibiotic triple therapy in FD patients—likely mediated by the mucosal-protective and anti-inflammatory properties of Weisu granules and berberine—may occur independently of bacterial clearance.

**Table 3:** Correlation between *Helicobacter pylori* eradication and symptom improvement.

	Successful eradication	Eradication failure	*P*-value
Markedly effective	12	4	1.000
Effective	15	6	
Ineffective	8	3	

### Incidence of adverse reactions and compliance

As shown in Table 4, 4 patients (8.3%; 4/48) experienced adverse reactions. The most common adverse events were dry mouth (2 cases), abdominal distension (1 case), and nausea (1 case). All adverse events were reported by female patients. Among these, 2 patients had undergone two prior eradication failures, and 2 had experienced three prior eradication failures. All adverse reactions were mild and resolved without intervention.

**Table 4:** Incidence of adverse events.

Symptoms	*N*	Gender	Age	BMI	Treatment times	Treatment continuation	Eradication
Dry mouth	2	F	21	17.9	3	Y	Y
		F	57	25.4	2	Y	N
Nausea	1	F	54	17.8	3	Y	N
Abdominal distension	1	F	38	24.8	2	N	N
Total	4						

BMI, body mass index (kg/m^2^).

The overall medication compliance rate for the 48 patients was 91.67%. Four patients exhibited a compliance rate of less than 80%: three failed to adhere to the prescribed dosing frequency, and one discontinued the regimen entirely due to an adverse event (abdominal distension). In the latter case, a follow-up assessment 4 weeks later confirmed *H. pylori* eradication failure.

## Discussion

**Figure 2:**
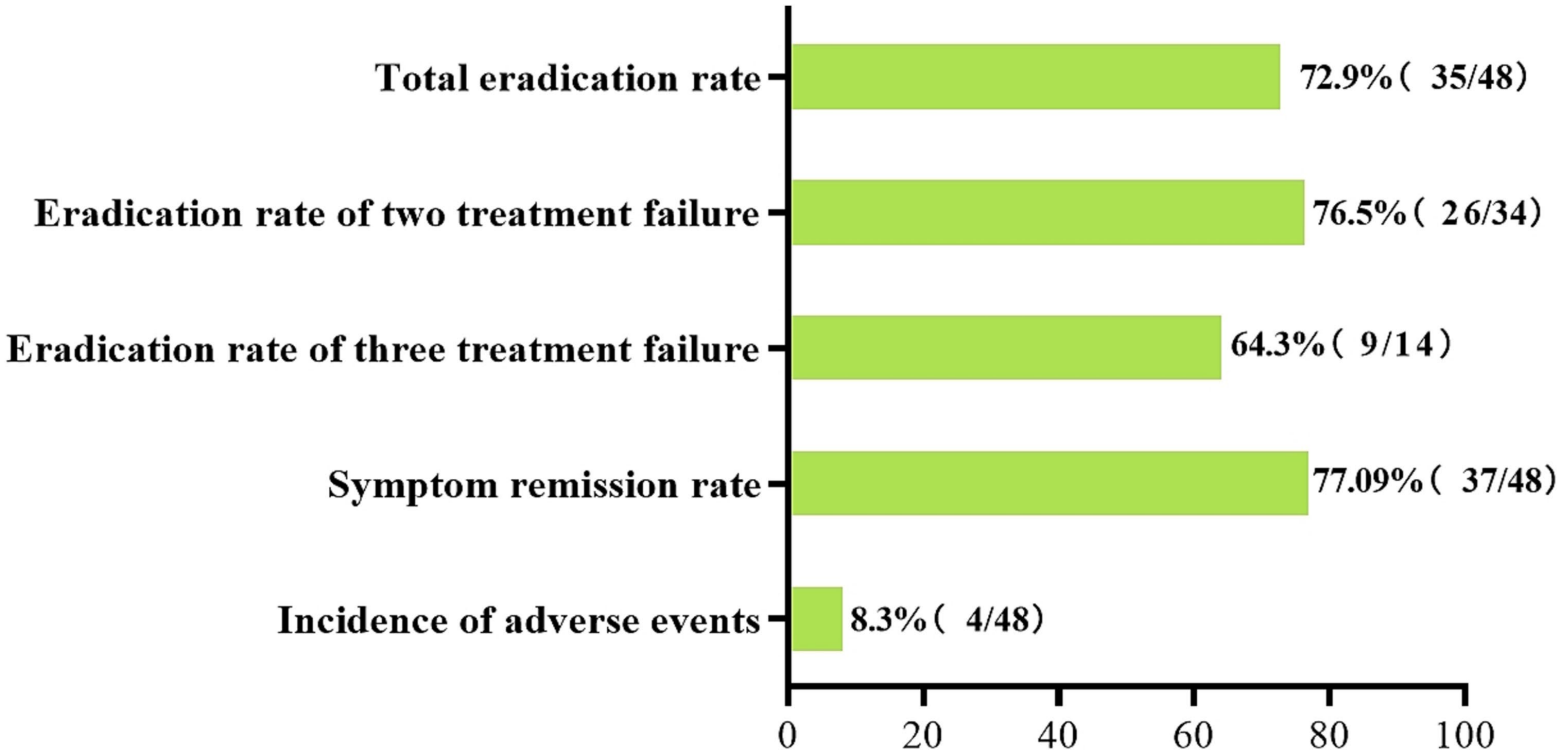
Total eradication rate, symptom relief rate, and incidence of adverse events.

To the best of our knowledge, this is the first observational study to evaluate the efficacy of a complete non-antibiotic regimen as a rescue treatment for patients with *H. pylori* eradication failure and FD. Our findings reveal an overall eradication rate of 72.9% and a clinical symptom relief rate of 77.08% with non-antibiotic triple therapy, alongside an 8.3% incidence of adverse events. This study confirms the potential of non-antibiotic triple therapy as an alternative for managing standard antibiotic-resistant *H. pylori* infection in FD patients, with high tolerability and compliance, as depicted in Figure 2.

As the efficacy of the current recommended first-line bismuth-based quadruple therapy has gradually decreased, the proportion of patients experiencing multiple eradication failures has increased (21, 22). Even with drug sensitivity testing for rescue treatment, antibiotics face significant challenges (23). In China, the resistance rate of *H. pylori* to amoxicillin is generally low (0–5%) (24). Consequently, several studies have suggested that high-dose dual therapy, combining a PPI or a potassium-competitive acid blocker (P-CAB) with amoxicillin, represents a promising and safe treatment option (25–27). However, regional variations are significant. In the present study, antibiotics were not used as a rescue treatment because the amoxicillin resistance rate of *H. pylori* in Guizhou Province has been reported as high as 13.27% (28). The disparity between national figures and Guizhou’s resistance rate may be attributed to the widespread and often unregulated use of amoxicillin for respiratory and gastrointestinal infections in this region, leading to increased primary resistance. This elevated local resistance suggests that standard dual therapies may be less effective in our patient population, warranting exploration of non-antibiotic alternatives such as Weisu granules, berberine, and Bio-Three. Furthermore, a meta-analysis showed that both high-dose dual therapy and guideline-recommended rescue therapies achieved comparable efficacy (81.3% vs. 81.5%, relative risk [RR] 1.00, 95% confidence interval [CI] 0.93–1.08) (29). Additionally, the patients included in this study had previously failed to respond to antibiotic therapy on two or more occasions.

A systematic review and meta-analysis have indicated that traditional Chinese medicine combined with western medicine yields higher eradication and symptom relief rates compared to western medicine alone, with acceptable safety profiles (30). Consequently, there is increasing attention on the eradication efficacy of non-antibiotic drugs against *H. pylori*. The total eradication rate of *H. pylori* with the non-antibiotic triple therapy in our study (72.9%) was comparable to or even higher than the efficacy of bismuth-based quadruple therapy (BQT) as a rescue treatment in previous meta-analyses (23), suggesting its potential as an alternative for standard antibiotic-resistant *H. pylori* infection in our region. Subgroup analyses of patients who had previously failed eradication twice and three times revealed eradication rates of 76.5 and 64.3%, respectively. Although the numerical rate was higher in the double-failure group, the difference was not statistically significant (*p* = 0.613). These results suggest that non-antibiotic therapy maintains comparable efficacy regardless of the number of prior antibiotic treatment failures. This highlights its potential as a versatile rescue treatment for refractory *H. pylori* infections in our region, even for the most difficult-to-treat patients who have failed multiple conventional regimens.

Moreover, demographic and clinical data analysis demonstrated that this regimen’s efficacy was unaffected by factors such as gender, smoking or drinking habits, BMI, previous treatment frequency, and family history of gastric cancer, suggesting its suitability for patients with diverse *H. pylori* eradication histories. Thus, in the face of high antibiotic resistance rates, the lack of antibiotic sensitivity testing, and patients with multiple comorbidities unsuitable for various antibiotics, the non-antibiotic triple therapy utilized in this study represents an effective alternative for patients with *H. pylori* eradication failure. However, given the small sample size and the single-arm, retrospective design of this study, further multicenter, prospective randomized controlled trials may be necessary to validate the regimen’s effectiveness.

The pathogenesis of FD involves multiple factors, including *H. pylori* infection (4). Evidence suggests that eradicating *H. pylori* can lead to improvements in FD symptoms (6). Numerous studies have demonstrated that Chinese herbal medicines or probiotics used in *H. pylori* treatment can not only enhance eradication rates but also alleviate FD symptoms and reduce adverse reactions (17, 30, 31).

A multicenter randomized controlled trial (RCT) conducted in Sichuan Province, China, compared the efficacy of a 14-day high-dose dual therapy (esomeprazole 20 mg g.i.d + amoxicillin 750 mg g.i.d) with standard bismuth quadruple therapy. The symptom relief rates were notable: 57.9% vs. 53.3% were markedly effective; 18.5% vs. 19.8% were effective; 2.8% vs. 4% were ineffective. The combined symptom relief rate was 76.4% (25). Encouragingly, in our study, the total clinical symptom relief rate was 77.08% (markedly effective 33.33% + effective 43.75%), slightly higher than that of the high-dose dual therapy and bismuth quadruple therapy in the aforementioned study. This indicates the effectiveness of our regimen in alleviating symptoms in FD patients with *H. pylori* infection. Regarding symptom relief, the relief rates for patients with two prior failures (79.41%) and three prior failures (71.43%) were numerically different; however, this difference was not statistically significant (*p* > 0.05). Consequently, these findings suggest that the clinical efficacy of the non-antibiotic regimen in alleviating symptoms is consistent and comparable across both groups, regardless of prior eradication attempts.

Furthermore, our analysis of the correlation between *H. pylori* eradication and FD symptom improvement found that successful eradication did not significantly improve FD symptoms compared to patients without eradication. This could be attributed to the small sample size of our study and the multifactorial nature of FD pathogenesis, which extends beyond *H. pylori* (4). Further confirmation through well-designed prospective large-sample studies is warranted.

Poor patient compliance is a significant contributor to treatment failure, with adverse events being a key deterrent (32). In our study, the incidence of adverse reactions was only 8.3%, with all events being mild and self-resolving. This incidence was lower than that with the standard antibiotic bismuth quadruple regimen and comparable to that with high-dose dual therapy (33). Patient compliance was generally good, with only four out of 48 patients taking less than 80% of the medication. Most patients tolerated the regimen well, with only one patient discontinuing treatment due to adverse reactions. Thus, our non-antibiotic therapy represents a relatively safe alternative for rescue treatment.

Our study is subject to several limitations that warrant careful consideration when interpreting the results. First and foremost, the study utilizes a retrospective, single-arm design without a control group. This design inherently limits our ability to draw definitive causal inferences about the intervention’s efficacy. This is particularly critical regarding the secondary endpoint of symptom improvement; given that Functional Dyspepsia is characterized by a fluctuating clinical course and is highly susceptible to the placebo effect, the symptom relief observed in this cohort cannot be unequivocally attributed solely to the pharmacological effects of the non-antibiotic regimen. Second, the sample size (*N* = 48) is relatively small. While sufficient for an exploratory, hypothesis-generating analysis, it limits the study’s statistical power and restricts the generalizability of the findings to the broader population of patients with refractory *H. pylori* infection. The small sample size also necessitates caution when interpreting subgroup analyses (e.g., comparing outcomes between patients with 2 versus 3 prior failures), as these findings may lack statistical robustness.

Third, we acknowledge the potential influence of heterogeneity in prior treatment exposure. The participants had failed varying combinations of antibiotic regimens before enrollment. This heterogeneity in prior antibiotic exposure could lead to diverse baseline variations in gut microbiota composition and bacterial resistance profiles, acting as a potential confounding factor that was not controlled for in this analysis. Finally, the study was conducted at two centers within a single region (Guizhou Province). Consequently, regional factors, including specific dietary habits and local *characteristics of H. pylori strains*, may influence the external validity of the results. Future research should prioritize multi-center, randomized controlled trials with larger sample sizes and long-term follow-up to validate these preliminary findings and rigorously assess the clinical utility of this non-antibiotic approach.

## Conclusion

In summary, the non-antibiotic regimen holds promise as an effective alternative for patients with FD who are afflicted by standard antibiotic-resistant *H. pylori* infection, demonstrating notable rates of *H. pylori* eradication and symptom improvement. In contrast to antibiotic therapies, non-antibiotic approaches offer multifaceted, multi-target antibacterial effects, which are less prone to inducing drug resistance. They obviate the need to consider prior antibiotic exposure or conduct antibiotic sensitivity tests and entail a lower incidence of adverse reactions. Additionally, non-antibiotic interventions may reduce inflammation and facilitate mucosal repair. Future investigations should examine their underlying mechanisms or conduct multi-center, large-scale, randomized controlled clinical trials to validate their long-term efficacy and assess their role in current treatment strategies.

## Data Availability

The original contributions presented in the study are included in the article/Supplementary material, further inquiries can be directed to the corresponding author.

## References

[1] Malfertheiner P Megraud F Rokkas T Gisbert JP Liou J-M Schulz C . Management of *Helicobacter pylori* infection: the Maastricht VI/Florence consensus report. Gut. (2022) 71:1724–62. doi: 10.1136/gutjnl-2022-327745 35944925

[2] Li Y Choi H Leung K Jiang F Graham DY Leung WK. Global prevalence of *Helicobacter pylori* infection between 1980 and 2022: a systematic review and meta-analysis. Lancet Gastroenterol Hepatol. (2023) 8:553–64. doi: 10.1016/S2468-1253(23)00070-5, 37086739

[3] Ford AC Yuan Y Moayyedi P. *Helicobacter pylori* eradication therapy to prevent gastric cancer: systematic review and meta-analysis. Gut. (2020) 69:2113–21. doi: 10.1136/gutjnl-2020-320839, 32205420

[4] Talley NJ Ford AC. Functional dyspepsia. N Engl J Med. (2015) 373:1853–63. doi: 10.1056/NEJMra1501505, 26535514

[5] Bercík P De Giorgio R Blennerhassett P Verdú EF Barbara G Collins SM. Immune-mediated neural dysfunction in a murine model of chronic *Helicobacter pylori* infection. Gastroenterology. (2002) 123:1205–15. doi: 10.1053/gast.2002.36024, 12360482

[6] Ford AC Tsipotis E Yuan Y Leontiadis GI Moayyedi P. Efficacy of *Helicobacter pylori* eradication therapy for functional dyspepsia: updated systematic review and meta-analysis. Gut. (2022) 71:gutjnl-2021-326583. doi: 10.1136/gutjnl-2021-326583 35022266

[7] Kang SJ Park B Shin CM. *Helicobacter pylori* eradication therapy for functional dyspepsia: a meta-analysis by region and *H. pylori* prevalence. J Clin Med. (2019) 8:1324. doi: 10.3390/jcm8091324, 31466299 PMC6780123

[8] Zhou L Lu H Song Z Lyu B Chen Y Wang J . 2022 Chinese national clinical practice guideline on *Helicobacter pylori* eradication treatment. Chin Med J. (2022) 135:2899–910. doi: 10.1097/CM9.0000000000002546, 36579940 PMC10106216

[9] Chey WD Leontiadis GI Howden CW Moss SF. ACG clinical guideline: treatment of *Helicobacter pylori* infection. Am J Gastroenterol. (2017) 112:212–39. doi: 10.1038/ajg.2016.563, 28071659

[10] Malfertheiner P Camargo MC El-Omar E Liou J-M Peek R Schulz C . *Helicobacter pylori* infection. Nat Rev Dis Primers. (2023) 9:20. doi: 10.1038/s41572-023-00431-8 PMC11558793 37081005

[11] Malfertheiner P Megraud F O'Morain CA Gisbert JP Kuipers EJ Axon AT . Management of *Helicobacter pylori* infection-the Maastricht V/Florence consensus report. Gut. (2017) 66:6–30. doi: 10.1136/gutjnl-2016-312288, 27707777

[12] Hu R Song T Liu JJ Jia F Wen W. Effects of gastric Su granules combined with quadruple therapy on serum inflammatory factors, gastrointestinal hormones, and quality of life in patients with *Helicobacter pylori*-associated peptic ulcer. Adv Mod Biomed. (2021) 21:2675–2678, 2707. doi: 10.13241/j.cnki.pmb.2021.14.016

[13] Tan FR Wen B Sun L. Study on the effect of gastric Su granules combined with triple bactericidal therapy in treating patients with *Helicobacter pylori* infection. Mod Diagn Treatment. (2023) 34:650–653, 656. (In Chinese)

[14] Song D Hao J Fan D. Biological properties and clinical applications of berberine. Front Med. (2020) 14:564–82. doi: 10.1007/s11684-019-0724-6, 32335802

[15] Shen Y Zou Y Chen X Li P Rao Y Yang X . Antibacterial self-assembled nanodrugs composed of berberine derivatives and rhamnolipids against *Helicobacter pylori*. J Control Release. (2020) 328:575–86. doi: 10.1016/j.jconrel.2020.09.025, 32946873

[16] Chen XX Chen YX Bi HX Zhao X Zhang LF Liu JY . Efficacy and safety of triple therapy containing berberine hydrochloride, amoxicillin, and rabeprazole in the eradication of *Helicobacter pylori*. J Dig Dis. (2022) 23:568–76. doi: 10.1111/1751-2980.13146, 36415112 PMC10107123

[17] Ji J Yang H. Using probiotics as supplementation for *Helicobacter pylori* antibiotic therapy. Int J Mol Sci. (2020) 21:1136. doi: 10.3390/ijms21031136, 32046317 PMC7037652

[18] Yuan L Yang C Han Y Yang F Tu H. Beyond antibiotics: probiotics as a promising ally against *Helicobacter pylori*. Front Pharmacol. (2025) 16:1620870. doi: 10.3389/fphar.2025.1620870 40717984 PMC12290408

[19] Ariyoshi T Hagihara M Takahashi M Mikamo H. Effect of *Clostridium butyricum* on gastrointestinal infections. Biomedicine. (2022) 10:483. doi: 10.3390/biomedicines10020483 PMC8962260 35203691

[20] Zhang J Guo J Li D Chen M Liu J Feng C . The efficacy and safety of clostridium butyricum and *Bacillus coagulans* in *Helicobacter pylori* eradication treatment: An open-label, single-arm pilot study. Medicine (Baltimore). (2020) 99:e22976. doi: 10.1097/MD.0000000000022976, 33157939 PMC7647598

[21] Zhong Z Zhang Z Wang J Hu Y Mi Y He B . A retrospective study of the antibiotic-resistant phenotypes and genotypes of *Helicobacter pylori* strains in China. Am J Cancer Res. (2021) 11:5027–37. doi: 10.3760/cma.j.issn.0376-2491.2022.22.102 34765309 PMC8569369

[22] Zou Y Qian X Liu X Song Y Song C Wu S . The effect of antibiotic resistance on *Helicobacter pylori* eradication efficacy: a systematic review and meta-analysis. Helicobacter. (2020) 25:e12714. doi: 10.1111/hel.12714, 32533599

[23] de Moraes Andrade PV Monteiro YM Chehter EZ. Third-line and rescue therapy for refractory *Helicobacter pylori* infection: a systematic review. World J Gastroenterol. (2023) 29:390–409. doi: 10.3748/wjg.v29.i2.390, 36687120 PMC9846933

[24] Chen J Li P Huang Y Guo Y Ding Z Lu H. Primary antibiotic resistance of *Helicobacter pylori* in different regions of China: a systematic review and meta-analysis. Pathogens. (2022) 11:786. doi: 10.3390/pathogens11070786, 35890031 PMC9316315

[25] Shen C Li C Lv M Dai X Gao C Li L . The prospective multiple-Centre randomized controlled clinical study of high-dose amoxicillin-proton pump inhibitor dual therapy for *H. pylori* infection in Sichuan areas. Ann Med. (2022) 54:426–35. doi: 10.1080/07853890.2022.2031269, 35098820 PMC8812792

[26] Hu J Mei H Su NY Sun WJ Zhang DK Fan LL . Eradication rates of *Helicobacter pylori* in treatment-naive patients following 14-day vonoprazan-amoxicillin dual therapy: a multicenter randomized controlled trial in China. Helicobacter. (2023) 28:e12970. doi: 10.1111/hel.12970, 37160689

[27] Gao W Teng G Wang C Xu Y Li Y Cheng H. Eradication rate and safety of a "simplified rescue therapy": 14-day vonoprazan and amoxicillin dual regimen as rescue therapy on treatment of *Helicobacter pylori* infection previously failed in eradication: a real-world, retrospective clinical study in China. Helicobacter. (2022) 27:e12918. doi: 10.1111/hel.12918, 35877765 PMC9542484

[28] Wu FC Wang Q Zhu J Hu Y Pan K Jiang Q . Drug susceptibility and pbp1 diversity analysis of 351 *Helicobacter pylori* strains in Guiyang. Chinese J Hum Vet Dis. (2019) 35:587–93. doi: 10.3969/j.issn.1002-2694.2019.00.092

[29] Gao CP Zhou Z Wang JZ Han SX Li LP Lu H. Efficacy and safety of high-dose dual therapy for *Helicobacter pylori* rescue therapy: a systematic review and meta-analysis. J Dig Dis. (2016) 17:811–9. doi: 10.1111/1751-2980.12432, 27977071

[30] Zhong MF Li J Liu XL Gong P Zhang XT. TCM-based therapy as a rescue therapy for re-eradication of *Helicobacter pylori* infection: a systematic review and meta-analysis. Evid Based Complement Alternat Med. (2022) 2022:5626235. doi: 10.1155/2022/5626235 35251209 PMC8894008

[31] Yao G Fan X Lu D. Efficacy and safety of probiotic-supplemented bismuth quadruple therapy for the treatment of *Helicobacter pylori* infection: a systematic review and meta-analysis. J Int Med Res. (2023) 51:3000605231203841. doi: 10.1177/03000605231203841, 37848344 PMC10586011

[32] Cortés P Nelson AD Bi Y Stancampiano FF Murray LP Pujalte GGA . Treatment approach of refractory *Helicobacter pylori* infection: a comprehensive review. J Prim Care Community Health. (2021) 12:21501327211014087. doi: 10.1177/21501327211014087, 33949229 PMC8114244

[33] Liu C Wang Y Shi J Zhang C Nie J Li S . The status and progress of first-line treatment against *Helicobacter pylori* infection: a review. Ther Adv Gastroenterol. (2021) 14:1756284821989177. doi: 10.1177/1756284821989177, 34262609 PMC8243100

