# Supplementary material for: Novel non-antibiotic triple therapy for Helicobacter pylori-positive functional dyspepsia patients resistant to conventional antibiotic treatments: an exploratory pilot study
Source: Front Med (Lausanne). 2026 Feb 11;13:1759043. doi: 10.3389/fmed.2026.1759043 (PMC12932454; doi:10.3389/fmed.2026.1759043)
Supplement: Supplementary file 1 [file Table_1.docx]

**Table S1. Non-Antibiotic Triple Therapy Regimen (14-Day Course)**

| Medication | Unit Strength | Dosage per Administration | Frequency | Total Quantity per Patient |
| --- | --- | --- | --- | --- |
| Weisu Granules | 5 g/ bag | 5 g (1 bag) | t.i.d. | 42 bags |
| Berberine Hydrochloride | 0.1 g/ tablet | 0.3 g (3 tablets) | t.i.d. | 126 tablets |
| Bio-Three | 200 mg/ tablet | 400 mg (2 tablets) | t.i.d. | 84 tablets |
